# MICOS and MIMAS, multifunctional assemblies linking mitochondrial biogenesis, architecture, and function

**DOI:** 10.1002/pro.70653

**Published:** 2026-05-24

**Authors:** Patrick Horten, Kuo Song, Nikolaus Pfanner, Heike Rampelt

**Affiliations:** ^1^ Institute of Biochemistry and Molecular Biology, ZBMZ, Faculty of Medicine University of Freiburg Freiburg Germany; ^2^ Faculty of Biology University of Freiburg Freiburg Germany; ^3^ CIBSS Centre for Integrative Biological Signalling Studies University of Freiburg Freiburg Germany

**Keywords:** cristae, membrane organization, metabolism, mitochondria, respiratory chain

## Abstract

Mitochondrial cristae architecture is central for optimal oxidative phosphorylation and a healthy mitochondrial physiology. The intricate architecture of the inner mitochondrial membrane relies on protein complexes that compartmentalize the membrane by imposing membrane curvature, forming membrane contact sites or membrane subdomains, regulating the partitioning of mitochondrial proteins between the different subcompartments and thereby enabling functional asymmetry, and by governing membrane dynamics. Studies in recent years have expanded our understanding of the machineries and mechanisms underlying the manifold functions of the inner membrane. This review focuses on the mitochondrial contact site and cristae organizing system (MICOS), a protein complex that stabilizes the narrow entry gates of cristae, and on a novel inner membrane megacomplex, the mitochondrial multifunctional assembly (MIMAS), as well as on their roles in organizing the inner membrane.

## 1 INTRODUCTION

Mitochondria host a large variety of metabolic pathways, most prominent among them oxidative phosphorylation which provides the vast majority of the cellular ATP. Respiration and ATP synthesis take place in the inner mitochondrial membrane whose architecture is organized around optimal functioning of oxidative phosphorylation (Cogliati et al., 2016; Klecker & Westermann, 2021; Pánek et al., 2020; Pfanner et al., 2019). The inner mitochondrial membrane ranges among the most protein‐dense membranes of eukaryotic cells; it is topologically highly complex and comprises physically and functionally distinct subcompartments. It is folded into the characteristic cristae, sheet‐like or tubular membranes that host the complexes of the respiratory chain and the F_1_F_o_‐ATP synthase. The cristae membranes are continuous with the planar domains of the inner membrane, the inner boundary membrane, that is located underneath the outer membrane. However, the connections between inner boundary membrane and cristae impose significant bottlenecks for the movement of proteins and complexes. These so‐called crista junctions are narrow neck‐like membrane stalks whose diameter is typically around 20–30 nm. The complex inner membrane architecture relies on protein complexes that bend and/or scaffold the membrane (Colina‐Tenorio et al., 2020; Eramo et al., 2020; Kühlbrandt, 2019; Mukherjee et al., 2021; Zick et al., 2009). Moreover, the asymmetric distribution of protein complexes between the inner boundary membrane and cristae implies the existence of mechanisms that regulate the lateral segregation of proteins in this intricate landscape. The mitochondrial contact site and cristae organizing system (MICOS) and the mitochondrial multifunctional assembly (MIMAS) both combine roles in membrane compartmentalization with regulatory functions in mitochondrial biogenesis, architecture, and metabolism.

### 1.1 The mitochondrial multifunctional assembly MIMAS, a novel principle of membrane protein segregation

**FIGURE 1:**
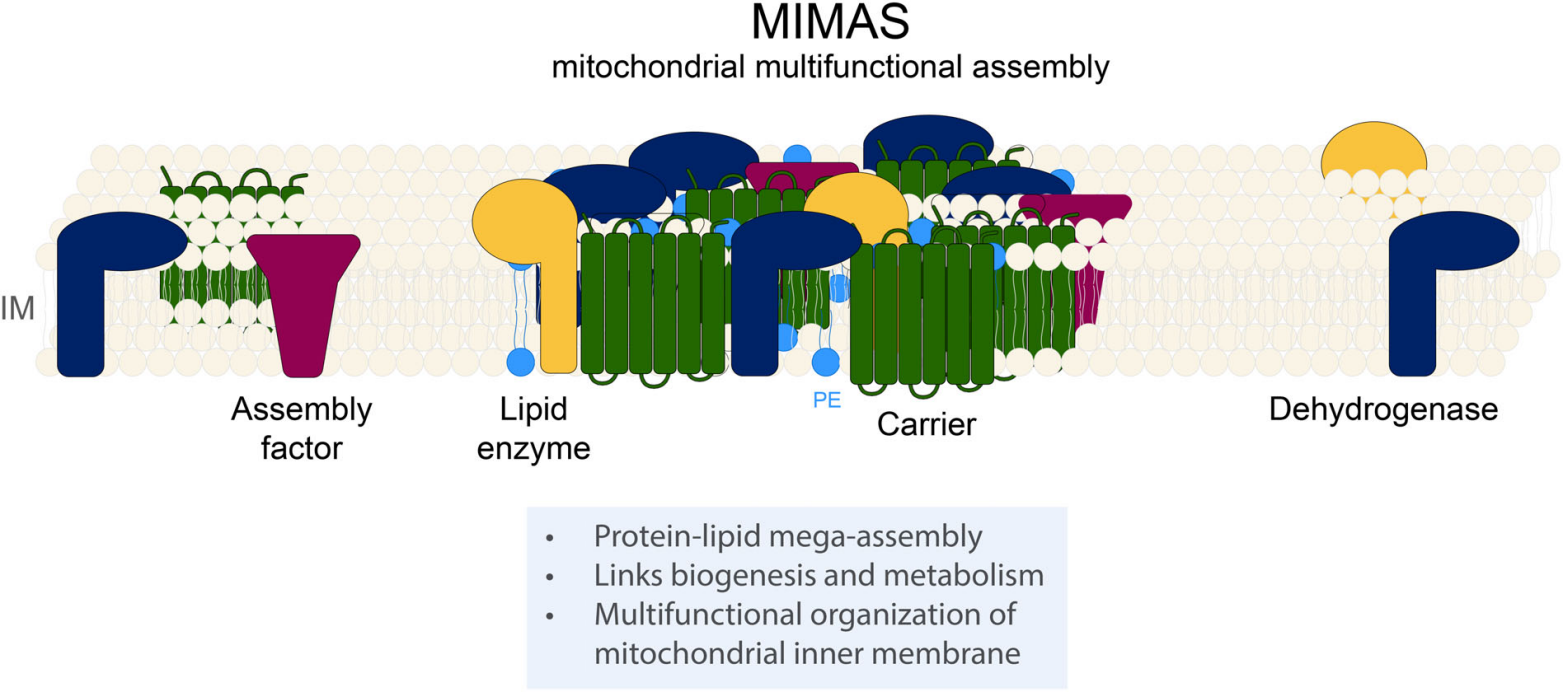
The mitochondrial multifunctional assembly (MIMAS) includes inner membrane‐localized proteins from different functional groups: mitochondrial metabolite carriers, dehydrogenases, respiratory chain assembly factors, and phospholipid synthesis enzymes. IM, inner membrane.

The inner mitochondrial membrane is home to many well‐known protein complexes, among them the respiratory chain complexes, the F_1_F_o_‐ATP synthase, protein translocase complexes, MICOS, and the prohibitin rings. However, there are many additional inner membrane proteins that are not assembled into any of these complexes. For some of these proteins, smaller, often transient complexes have been described, or they have been assumed to diffuse freely in the inner membrane (Morgenstern et al., 2017; Schulte et al., 2023). Recent work has led to the surprising discovery that many of these supposedly solitary proteins are organized in a novel megacomplex termed MIMAS for mitochondrial multifunctional assembly (Horten et al., 2024). Supported by the high‐resolution yeast mitochondrial complexome dataset MitCOM (Schulte et al., 2023), the study found that proteins from several functional groups are associated in a ~3 MDa complex. MIMAS components include a multitude of biogenesis and assembly factors for the respiratory chain, mitochondrial metabolite carriers, and enzymes involved in bioenergetics and in the synthesis of phospholipids (Horten et al., 2024) (Figure 1). MIMAS predominantly contains proteins that are anchored in the inner membrane but also includes, for example, the intermembrane space‐localized heme lyases for cytochrome *c* and *c1* that were previously reported as membrane‐associated but do not appear to contain hydrophobic motifs (Horten et al., 2024). It remains to be shown whether matrix or outer membrane proteins can also associate with MIMAS. For most MIMAS components analyzed, their proportion in MIMAS versus other complexes was estimated at up to 20%–40%. The bulk of MIMAS appears to consist of highly abundant metabolite carriers such as the ADP‐ATP carrier as well as of dehydrogenases (Figure 1). In contrast, subunits of the respiratory chain and other well‐known complexes are not associated with MIMAS to a significant degree.

The enzyme responsible for mitochondrial production of phosphatidylethanolamine (PE), Psd1 (Horvath & Daum, 2013), is a MIMAS component and its activity is required for MIMAS stability, indicating that many interactions within MIMAS are PE‐dependent. This raises the interesting question whether there are differences in the levels or acyl chain composition of PE at MIMAS versus other submitochondrial regions.

Despite its surprising characteristics and composition, MIMAS behaves like established mitochondrial membrane complexes upon detergent solubilization; it can be biochemically isolated and its proteome has been analyzed (Horten et al., 2024). Crosslinking in intact mitochondria demonstrated a close proximity of MIMAS components from different functional groups. Moreover, the cytochrome *c* oxidase (complex IV) assembly factor Rcf1 (Garlich et al., 2017; Strogolova et al., 2012; Vukotic et al., 2012) associates with both MIMAS and the respiratory chain (Horten et al., 2024). MIMAS thus participates in the assembly of the complex IV subunit Cox13, which depends on Rcf1. Rcf1 can be shifted between MIMAS and the respiratory chain by differential point mutations, highlighting the specificity of the interactions (Horten et al., 2024).

Interestingly, some MIMAS components are among the proteins identified as being localized in the vicinity of either the prohibitin complex or the peptide exit tunnel of mitochondrial ribosomes (Kohler et al., 2023). The study found that via these interactions, mitochondrially encoded subunits of the respiratory chain are subject to triage for either folding and assembly or degradation. The findings raise the interesting question if MIMAS is recruited to these triage points and contributes biogenesis factors for efficient assembly.

We propose that MIMAS integrates metabolic cues with protein complex assembly and phospholipid biosynthesis. Importantly, segregating a variety of proteins as part of MIMAS constitutes a mechanism of membrane compartmentalization that may serve to collectively regulate their submitochondrial localization or activities or to protect them from unwanted interactions as well as degradation. In light of the extraordinary protein density of the inner mitochondrial membrane (Horvath & Daum, 2013; Krebs et al., 1979; Sperka‐Gottlieb et al., 1988), it is conceivable that MIMAS helps to control the behavior and fate of otherwise freely diffusible proteins and thus represents a novel membrane organizing paradigm.

### 1.2 MICOS, the mitochondrial contact site and cristae organizing system

**FIGURE 2:**
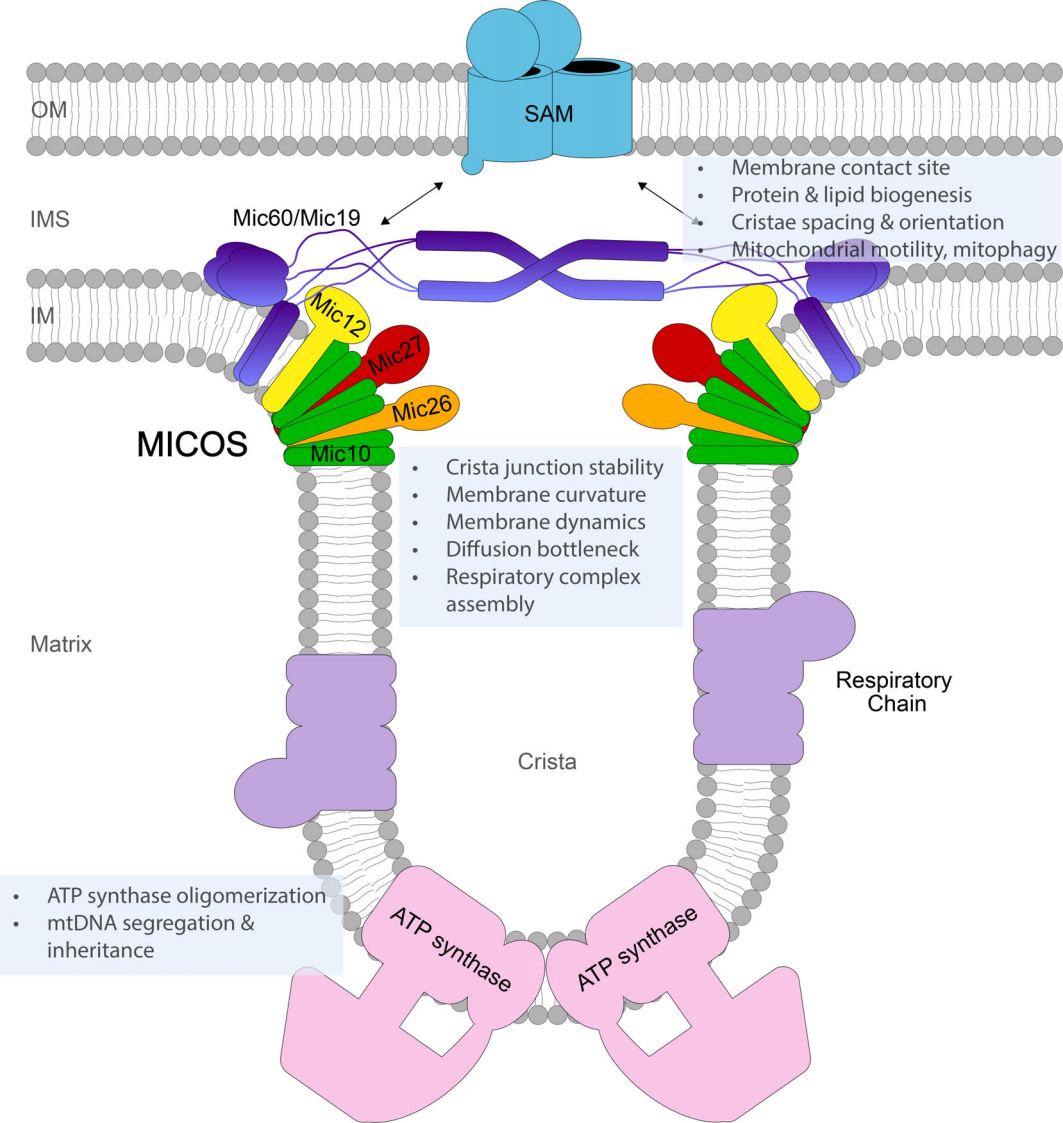
Diverse functions of the mitochondrial contact site and cristae organizing system (MICOS). The inner membrane complex MICOS is required for the stability of crista junctions, but performs many additional roles in mitochondria. OM, outer membrane; IMS, intermembrane space; IM, inner membrane; SAM, sorting and assembly machinery.

MICOS is a widely conserved inner membrane protein complex at the entrance to cristae, comprising six subunits in fungi and seven in vertebrates (Figure 2) (Colina‐Tenorio et al., 2020; Daumke & van der Laan, 2025; Eramo et al., 2020; Guarani et al., 2015; Harner et al., 2011; Hoppins et al., 2011; Mukherjee et al., 2021; Ott et al., 2012; von der Malsburg et al., 2011). In yeast its molecular mass is over 3.5 MDa (Schulte et al., 2023). The core components Mic60 and Mic10 are the evolutionarily oldest ones, with Mic60 being present already in α‐proteobacteria, and they are associated with the presence of mitochondrial cristae or intracellular membranes (in α‐proteobacteria) (Huynen et al., 2016; Kaurov et al., 2018; Muñoz‐Gómez et al., 2015; Venkatraman et al., 2023). Although MICOS has emerged as a multifunctional hub with a multitude of regulatory and organizing roles, arguably its primary role is the stabilization of crista junctions. Mic60 and Mic10 are both required to induce the strong local membrane curvature at crista junctions, and Mic60 additionally forms contacts to the outer mitochondrial membrane (Barbot et al., 2015; Bohnert et al., 2015; Harner et al., 2011; Hessenberger et al., 2017; Ott et al., 2012; Tarasenko et al., 2017; von der Malsburg et al., 2011). Loss of either subunit results in the detachment of cristae membranes from the inner boundary membrane and the formation of aberrant cristae. MICOS‐deficient cells display significant defects in respiratory growth. Mic60 and Mic10 each form subcomplexes with non‐redundant functions. The MIC60 subcomplex includes Mic19 and, in vertebrates, the Mic19‐related protein Mic25, while the MIC10 subcomplex includes the auxiliary proteins Mic12 (Mic13 or QIL1 in mammals), Mic26 and Mic27, with Mic12/QIL1 bridging the two subcomplexes (Colina‐Tenorio et al., 2020; Daumke & van der Laan, 2025; Eramo et al., 2020; Mukherjee et al., 2021). The crucial role of MICOS for mitochondrial physiology is exemplified by the human pathologies either resulting directly from MICOS dysfunction or associated with dysregulation of MICOS subunits. Mutations in QIL1 cause a fatal mitochondrial encephalopathy with liver pathology (Gödiker et al., 2018; Guarani et al., 2016; Kishita et al., 2020; Zeharia et al., 2016). For mutations in Mic26 (also called APOO) two distinct pathologies were reported, an X‐linked mitochondrial disease with neuromuscular symptoms, as well as a fatal progeria (Benincá et al., 2021; Peifer‐Weiß et al., 2023). Moreover, Mic60 variants with mutations in their mitochondrial targeting sequence, resulting in impaired mitochondrial import, were described for Parkinson's patients and caused severe, dominant‐negative defects when expressed in Drosophila (Tsai et al., 2018).

Mic60 (in mammals also termed IMMT or mitofilin) is anchored to the inner membrane by an amino‐terminal transmembrane segment and exposes an elongated coiled‐coil domain as well as the signature mitofilin domain, a helical bundle, to the intermembrane space (Benning et al., 2025; Bock‐Bierbaum et al., 2022; Hessenberger et al., 2017; Nathanail et al., 2026; Rabl et al., 2009; Tarasenko et al., 2017). Its partner protein Mic19 (CHCHD3) and the additional vertebrate‐specific MICOS subunit Mic25 (CHCHD6) are coiled‐coil helix coiled‐coil helix (CHCH) domain proteins whose fold is stabilized by disulfide bonds, and as such are imported by the mitochondrial intermembrane space import and assembly (MIA) pathway (Darshi et al., 2012; Hessenberger et al., 2017; Sakowska et al., 2015; Ueda et al., 2019; van der Schans et al., 2026). Mic19 and Mic25 are the only MICOS components that lack transmembrane segments. They are anchored to the membrane by amino‐terminal myristoylation. Mic60 is present as a tetramer and induces membrane curvature via a lipid‐binding site that forms an amphipathic helix, a function that is required for crista junction stability (Hessenberger et al., 2017; Tarasenko et al., 2017). Mic19 binds to the mitofilin domain in a 1:1 stoichiometry and regulates both the oligomeric state and membrane‐binding of Mic60. One of the first functions discovered for the MIC60 subcomplex is the formation of membrane contact sites due to its ability to interact with outer membrane proteins. It binds Sam50, the core subunit of the sorting and assembly machinery (SAM) which is responsible for the biogenesis of β‐barrel proteins in the outer mitochondrial membrane (Bohnert et al., 2012; Darshi et al., 2011; Ganesan et al., 2024; Harner et al., 2011; Huynen et al., 2016; Muñoz‐Gómez et al., 2023; Ott et al., 2012). This interaction is very robust in mammalian cells and results in a stable supercomplex termed mitochondrial intermembrane space bridging complex (MIB) which comprises both MICOS and SAM. The architecture of the MIC60 subcomplex/Sam50 membrane contact site is still debated (Bock‐Bierbaum et al., 2022; Bohnert et al., 2012; Darshi et al., 2011, 2012; Körner et al., 2012; Nathanail et al., 2026; Ott et al., 2012; Tang et al., 2020; Ueda et al., 2019; Zerbes et al., 2012). Additional outer membrane interactors of the MIC60 subcomplex in yeast are Tom40 of the translocase of the outer membrane (TOM) and the VDAC (voltage‐dependent anion channel) homolog porin (Hoppins et al., 2011; von der Malsburg et al., 2011). The anchoring of the MIC60 subcomplex to the outer membrane may also play a role in stabilizing the architecture of crista junctions. By bringing specific interactors into close proximity, Mic60 supports efficient protein biogenesis of MIA substrates as well as of β‐barrel proteins in yeast, and of metabolite carriers in human cells (Bohnert et al., 2012; Callegari et al., 2019; Harner et al., 2011; von der Malsburg et al., 2011). Moreover, as a consequence of forming membrane contact sites, Mic60 facilitates the synthesis of PE in the outer membrane by the inner membrane‐anchored enzyme Psd1 (Aaltonen et al., 2016).

Intriguingly, recent models based on partial structures of Mic60 and Mic19 domains suggest that Mic60 tetramers span the crista junction and impose a diffusion barrier for soluble proteins while also controlling the crista junction diameter (Bock‐Bierbaum et al., 2022; Nathanail et al., 2026). The presence of a significant diffusion barrier at crista junctions aligns well with the recent finding that cristae are independent bioenergetic units with individual membrane potentials (Wolf et al., 2019). At the same time, a surprisingly dynamic behavior of cristae and crista junctions has been discovered in recent years (Harner et al., 2016; Kondadi et al., 2020; Kondadi & Reichert, 2024; Stephan et al., 2020).

The second MICOS core subunit, Mic10, is a small protein with two transmembrane segments of differing length connected by a very short loop in the mitochondrial matrix (Barbot et al., 2015; Bohnert et al., 2015). It is assumed that these characteristics give Mic10 a wedge‐like shape in the membrane, resulting in asymmetric displacement of lipids in the two membrane leaflets. Additionally, Mic10 forms oligomers via glycine‐containing motifs that mediate helix–helix interactions in membranes (Barbot et al., 2015; Bohnert et al., 2015; Kaurov et al., 2018; Stephan et al., 2024). Together, these properties render Mic10 a powerful inducer of membrane curvature following a similar mode of action as reticulons in the endoplasmatic reticulum. Mic10 oligomers are stabilized by binding of the mitochondrial signature phospholipid cardiolipin (Rampelt et al., 2018). Defects in Mic10 oligomerization disrupt MICOS function, highlighting the importance of Mic10‐mediated scaffolding of the strongly curved crista junction membranes (Barbot et al., 2015; Bohnert et al., 2015). In yeast, oligomerization of Mic10 is regulated antagonistically by the auxiliary MIC10 subcomplex components Mic26 and Mic27. While Mic27 stabilizes Mic10 oligomers, Mic26 has a destabilizing effect (Rampelt et al., 2018). The mammalian namesakes of these proteins, also known as APOO and APOOL, are not direct orthologs of the yeast proteins since the genes arose from independent duplication events (Huynen et al., 2016). Consequently, it is unclear how the mammalian Mic26 and Mic27 regulate Mic10 oligomers, but their loss selectively affects higher order assemblies of MICOS (Anand et al., 2020; Weber et al., 2013). Human cells lacking both subunits display significantly decreased cardiolipin levels as well as defects in the stability of inner membrane complexes such as the F_1_F_o_‐ATP synthase and respiratory chain supercomplexes (Anand et al., 2020). Since cardiolipin accumulation depends on respiratory chain assembly (Xu et al., 2019, 2021) and respiratory supercomplex stability depends on cardiolipin (Pfeiffer et al., 2003), it is still unclear what the direct effects of the double Mic26/Mic27 knockout are. The MICOS subunit Mic12/QIL1 connects the MIC10 and MIC60 subcomplexes (Guarani et al., 2015; Urbach et al., 2021; Zerbes et al., 2016).

### 1.3 MICOS, a functional and structural organizer of the inner mitochondrial membrane

Far from simply serving as a structural scaffold, MICOS components play regulatory and organizing roles in multiple aspects of mitochondrial physiology. As mentioned earlier, Mic60 promotes protein import via specific pathways and supports phospholipid biogenesis—processes that involve productive coordination of participants residing in different submitochondrial compartments. In *Trypanosoma brucei*, an organism that is evolutionarily very distant from both fungi and metazoa, MICOS is quite diverged and includes, among others, subunits without orthologs in opisthokonts, an unusual Mic60 ortholog and separate mitofilin domain proteins (Eichenberger et al., 2019; Kaurov et al., 2018; Sheikh et al., 2025). Despite these substantial differences, the trypanosome MICOS still supports mitochondrial protein import for specific substrates, highlighting the conserved role of MICOS as an organizer of multi‐compartment processes.

Mic10 performs a surprising function outside of MICOS. It interacts with dimers of the F_1_F_o_‐ATP synthase and stabilizes their oligomeric rows (Cadena et al., 2021; Rampelt et al., 2017, 2022). Dimers and oligomers of the ATP synthase are critical for forming the membrane curvature at cristae rims and tubular cristae and thus represent an opposing, but equally important force that determines the inner membrane architecture together with MICOS (Paumard et al., 2002; Strauss et al., 2008; Rabl et al., 2009; Horten & Rampelt, 2024). Mic10 variants that do not rescue the MICOS defect but interact with the ATP synthase were able to restore the decreased membrane potential of Mic10‐deficient mitochondria as well as the growth of *mic10*Δ yeast cells during adaptation from fermentative to respiratory metabolism (Rampelt et al., 2022). Since mitochondrial biogenesis and cristae biogenesis are strongly induced under these conditions, the novel regulatory function of Mic10 could serve to coordinate the membrane‐shaping activities of MICOS and ATP synthase. Interestingly, this interaction was found also in *Trypanosoma brucei* which expresses two Mic10 isoforms, only one of which was found to interact with the ATP synthase (Cadena et al., 2021).

The localization of MICOS at the entry gate of cristae membranes, potentially acting as a diffusion barrier or filter for soluble as well as membrane proteins, raises important questions for the biogenesis and proteostasis of cristae membrane protein complexes. The complexes of the respiratory chain are the most striking example of the many challenges that subunits of a protein complex face within the intricate architecture of the inner membrane. Respiratory chain complexes I, III and IV, and the ATP synthase are each composed of hydrophobic mitochondrially encoded subunits that are synthesized at mitochondrial ribosomes, as well as many nuclear‐encoded subunits that are imported from the cytosol and inserted into the inner boundary membrane. The subunits assemble consecutively with the help of numerous assembly factors that include chaperones, peptidases, and proteins that deliver and insert cofactors (Formosa et al., 2018; Ndi et al., 2018; Soto et al., 2012; Timón‐Gómez et al., 2018). Work in yeast has shown that early assembly steps for complexes III and IV are preferentially localized in the inner boundary membrane, whereas there is a shift toward cristae membranes for the late steps (Stoldt et al., 2019). These results indicate that at a late point during their maturation, assembly intermediates presumably travel through crista junctions into cristae membranes. While there are still many questions surrounding the precise architecture of MICOS at crista junctions, it seems safe to assume that assembly intermediates would have to pass through the immediate vicinity of MICOS. The question is, does MICOS function in this context as a passive scaffold that imposes an unspecific bottleneck on complexes, or is MICOS function more akin to a bouncer who selectively admits or even promotes the passage of some of those complexes that seek entry and rejects others? Two recent studies provide evidence for a specific supportive role of MICOS, showing that MICOS interacts with distinct intermediates of respiratory complexes III and IV and is important for efficient downstream assembly (Colina‐Tenorio et al., 2026; Zerbes et al., 2025). The studies demonstrate that MICOS deficiency does not cause global defects in inner membrane complex assembly, but rather impairs specific assembly steps that are connected to the assembly intermediates found to associate with Mic60. Mic60 appears to recruit these intermediates via the assembly factors maintaining them. In fact, Mic60 interacts with a large variety of respiratory chain assembly factors (Chojnacka et al., 2015; Colina‐Tenorio et al., 2026; Zerbes et al., 2025), and an unbiased interactome analysis revealed that Mic60 co‐isolates many MIMAS components (Colina‐Tenorio et al., 2026). Many of these interactions were sensitive to loss of Psd1 and to solubilization with Triton, either of which destabilizes MIMAS (Colina‐Tenorio et al., 2026; Horten et al., 2024). In contrast, a subset of assembly factor associations with Mic60 were resistant to these treatments and may therefore represent direct interactors of MICOS. These results indicate that, using different mechanisms (direct binding or interaction via MIMAS), Mic60 recruits many different assembly factors to MICOS, that is to contact sites and crista junctions. In this way, MICOS facilitates assembly steps, likely by enhancing the probability of productive interactions of intermediates and assembly factors, right in the areas that respiratory chain intermediates have to pass through in order to reach the cristae. Moreover, these findings suggest that one purpose of organizing various proteins in a dynamic reservoir such as MIMAS may indeed be to bulk‐regulate their submitochondrial localization or their environment in the membrane. Thus, the sequestration of respiratory chain assembly factors in MIMAS enables their recruitment to crista junctions via the interaction of MIMAS with MICOS. Many open questions remain: Does MICOS support the spatial segregation of intermediates into cristae membranes? Are some proteins or complexes prevented from passing through crista junctions by the action of MICOS? How is the repair of mature cristae‐localized complexes achieved?

MICOS also plays an important role in the segregation and inheritance of mitochondrial DNA which is organized as nucleoids (Itoh et al., 2013; Jakubke et al., 2021; Landoni et al., 2026; Li et al., 2016; Macuada et al., 2025; Yang et al., 2015). Upon MICOS disruption, nucleoids aggregate, mitochondria become fragmented, and selective retention of defective mtDNA in the yeast zygote is disrupted. The observed defects may be consequences of the altered cristae architecture of MICOS mutants, but MICOS may also contribute to nucleoid organization more directly. On the one hand, defective cristae architecture impairs mitochondrial dynamics, which likely plays a role in defective nucleoid inheritance (Hoppins et al., 2011; Itoh et al., 2013; Jakubke et al., 2021; Li et al., 2016; Ott et al., 2015; Rabl et al., 2009). On the other hand, MICOS has been reported to localize in proximity to nucleoids as well as the outer membrane Rho GTPase Miro that mediates mitochondrial motility via microtubules and actin (Itoh et al., 2013; Li et al., 2016; Li et al., 2021; López‐Doménech et al., 2018; Modi et al., 2019; Oeding et al., 2018; Qin et al., 2020; Shi et al., 2022; Tsai et al., 2017; Yang et al., 2015). Accordingly, MICOS may contribute actively to the segregation and distribution of mtDNA. Additionally, even without restoring cristae architecture, some MICOS defects in mitochondrial dynamics and nucleoid segregation can be rescued by simultaneous disruption of ATP synthase dimerization or by regulating the ATP synthase (Itoh et al., 2013; Rampelt et al., 2022).

The inner mitochondrial membrane is organized in unexpected ways on a higher level. The lamellar cristae of mammalian cells are parallel, quite regularly spaced and oriented in a fashion perpendicular to the mitochondrial surface, and MICOS complexes at crista junctions can be visualized as helical bands lining the mitochondrial periphery (Hoppins et al., 2011; Jans et al., 2013; Pape et al., 2020; Stoldt et al., 2019). Moreover, cristae are even aligned to each other in adjacent mitochondria, involving so far unidentified structures in the outer membrane termed inter‐mitochondrial junctions (Heine & Hood, 2020; Picard et al., 2015). The highly ordered appearance of native cristae architecture is especially striking when compared to mutants that cannot maintain it. For instance, loss of Mic60 or Mic10 in cultured human cells results in loss of lamellar cristae and formation of an expansive tube‐like membrane system (Stephan et al., 2020), and Mic60 downregulation in Drosophila leads to apparently stochastic reorientation of cristae (Wang et al., 2020). In addition to MICOS and SAM (forming the MIB complex), the dynamin‐related GTPase OPA1 and the ATP synthase, two well established cristae architecture determinants, as well as Miro proteins appear to regulate the organization and spacing of cristae (Modi et al., 2019; Stephan et al., 2020), but the nature of this membrane architecture is poorly understood. OPA1, in addition to driving inner membrane fusion, also regulates the shape of cristae and crista junctions, which is an important factor for the release of cytochrome *c* from the cristae lumen during apoptosis (Cipolat et al., 2006; Frezza et al., 2006; Glytsou et al., 2016; Scorrano et al., 2002; Yamaguchi et al., 2008). While MICOS dissociation has been shown during apoptosis (Große et al., 2016), cytochrome *c* release appears to depend more on OPA1 remodeling.

## 2 PERSPECTIVES

The many surprising insights of the past few years have made it clear that there are still new functional connections, interaction networks and even new principles for membrane compartmentalization left to discover in the field of inner mitochondrial membrane organization. New approaches are uncovering novel determinants and parameters of cristae architecture (Hassdenteufel et al., 2025; Medina et al., 2025; Zhang et al., 2026). Many open questions remain. What is the structure of MICOS and of the MIB complex at crista junctions and how do these complexes impact the movement of proteins between the mitochondrial subcompartments? How are spacing, orientation, and the dynamic behavior of cristae determined and regulated? How are these functions impacted and safeguarded under stress conditions? What is the physiological relevance of sequestering MIMAS proteins involved in metabolic as well as protein biogenesis processes? Is MIMAS conserved in mammalian mitochondria? How are membrane protein biogenesis and complex assembly balanced with phospholipid synthesis? How important is cristae biogenesis versus cristae remodeling in different organisms and cell types? These and more research questions will undoubtedly be addressed in the near future, continuously expanding our understanding of the complexity of mitochondrial membrane organization.

## AUTHOR CONTRIBUTIONS

**Heike Rampelt:** Conceptualization; writing – original draft; writing – review and editing; data curation; supervision; funding acquisition; visualization. **Kuo Song:** Data curation; writing – review and editing. **Patrick Horten:** Visualization; writing – review and editing. **Nikolaus Pfanner:** Conceptualization; funding acquisition; writing – review and editing.

## CONFLICT OF INTEREST STATEMENT

The authors declare no conflicts of interest.

## Data Availability

Data sharing not applicable to this article as no datasets were generated or analyzed during this study.
